# Supplementary material for: The specialized inner ear labyrinth of worm-lizards (Amphisbaenia: Squamata)
Source: PLoS One. 2024 Nov 14;19(11):e0312086. doi: 10.1371/journal.pone.0312086 (PMC11563369; doi:10.1371/journal.pone.0312086)
Supplement: Attachment — Submitted filename: PONE-D-24-09623.pdf [file pone.0312086.s003.pdf]

PLOS ONE

The unique inner ear labyrinth of worm-lizards (Amphisbaenia: Squamata)

--Manuscript Draft--

**Manuscript Number:**	PONE-D-24-09623
**Article Type:**	Research Article
**Full Title:**	The unique inner ear labyrinth of worm-lizards (Amphisbaenia: Squamata)
**Short Title:**	The innear ear of worm-lizards
**Corresponding Author:**	Juan Daza<br>Sam Houston State University<br>Villanova, PA UNITED STATES
**Keywords:**	CT scanning<br>Reptiles<br>Lizard<br>Worm-Lizard<br>Morphology
**Abstract:**	Amphisbaenians are the most specialized group of reptiles for dwelling in fossorial habitats. Studies of the morphology of the inner ear based on cast derived from High-resolution computed tomography (HRCT) data has become a widely used method to study this part of the ear system. Previous researchers also have used these 3D models to make inferences about the ecology of fossil taxa. Although squamates have been widely studied using these methods, amphisbaenians have received relatively low attention. In this paper we survey the anatomy of the inner and middle ear in these fossorial reptiles using HRCT models. We describe qualitatively and quantitatively (using 3D morphometrics) the anatomy of the inner ear. Amphisbaenians are very diverse in skull anatomy, especially the configuration of the snout, which reflects the correlation with digging modes. We demonstrate that the ear also exhibits a large diversity of configurations, which we propose to be adaptations to the environments where they live, especially soil types. Results from these analyses also allow to describe 11 new phylogenetic characters that can be of some utility in future phylogenetic analyses.
**Order of Authors:**	Juan Diego Daza
	Geneva E. Clark
	Alessandro Palci
	Rebecca J. Laver
	Cristian Hernandez-Morales
	Christian A. Perez-Martinez
	Patrick J. Lewis
	Monte L Thies
	Christopher J. Bell
	Christy A. Hipsley
	Johannes Müller
	Ricardo Montero
**Additional Information:**	
**Question**	**Response**
**Financial Disclosure**<br><br>Enter a financial disclosure statement that describes the sources of funding for the	Yes

Powered by Editorial Manager® and ProduXion Manager® from Aries Systems Corporation

work included in this submission. Review the submission guidelines for detailed requirements. View published research articles from *PLOS ONE* for specific examples.<br><br>This statement is required for submission and **will appear in the published article** if the submission is accepted. Please make sure it is accurate.<br><br>**Funded studies**<br>Enter a statement with the following details:<br>• Initials of the authors who received each award<br>• Grant numbers awarded to each author<br>• The full name of each funder<br>• URL of each funder website<br>• Did the sponsors or funders play any role in the study design, data collection and analysis, decision to publish, or preparation of the manuscript?<br><br>Did you receive funding for this work?	
Please add funding details.<br>as follow-up to "**Financial Disclosure**<br><br>Enter a financial disclosure statement that describes the sources of funding for the work included in this submission. Review the submission guidelines for detailed requirements. View published research articles from *PLOS ONE* for specific examples.<br><br>This statement is required for submission and **will appear in the published article** if the submission is accepted. Please make sure it is accurate.	The Carl Gans Fund, Wilson-Warner, James D. Long and the Biology Department Scholarships supported Geneva E. Clark. Sam Houston State University Enhancement Research Grant (2014) to Juan D. Daza, Patrick J. Lewis, Monte L. Thies supported obtaining data for this study and funds from the Jackson School of Geosciences at The University of Texas at Austin to Christopher J. Bell.

Powered by Editorial Manager® and ProduXion Manager® from Aries Systems Corporation

**Funded studies**<br>Enter a statement with the following details:<br>• Initials of the authors who received each award<br>• Grant numbers awarded to each author<br>• The full name of each funder<br>• URL of each funder website<br>• Did the sponsors or funders play any role in the study design, data collection and analysis, decision to publish, or preparation of the manuscript?<br><br>Did you receive funding for this work?"	
Please select the country of your main research funder (please select carefully as in some cases this is used in fee calculation).<br>as follow-up to "**Financial Disclosure**<br><br>Enter a financial disclosure statement that describes the sources of funding for the work included in this submission. Review the submission guidelines for detailed requirements. View published research articles from *PLOS ONE* for specific examples.<br><br>This statement is required for submission and **will appear in the published article** if the submission is accepted. Please make sure it is accurate.<br><br>**Funded studies**<br>Enter a statement with the following details:<br>• Initials of the authors who received each award<br>• Grant numbers awarded to each author<br>• The full name of each funder<br>• URL of each funder website<br>• Did the sponsors or funders play any role in the study design, data collection and analysis, decision to publish, or preparation of the manuscript?<br><br>Did you receive funding for this work?"	UNITED STATES - US
**Competing Interests**	The authors have declared that no competing interests exist.

Use the instructions below to enter a competing interest statement for this submission. On behalf of all authors, disclose any competing interests that could be perceived to bias this work—acknowledging all financial support and any other relevant financial or non-financial competing interests.<br><br>This statement is required for submission and **will appear in the published article** if the submission is accepted. Please make sure it is accurate and that any funding sources listed in your Funding Information later in the submission form are also declared in your Financial Disclosure statement.<br><br>View published research articles from *PLOS ONE* for specific examples.<br><br>**NO authors have competing interests**<br><br>Enter: *The authors have declared that no competing interests exist.*<br><br>**Authors with competing interests**<br><br>Enter competing interest details beginning with this statement:<br><br>*I have read the journal's policy and the authors of this manuscript have the following competing interests: [insert competing interests here]*<br><br>* typeset	
**Ethics Statement**<br><br>Enter an ethics statement for this submission. This statement is required if the study involved:<br><br>• Human participants<br>• Human specimens or tissue<br>• Vertebrate animals or cephalopods<br>• Vertebrate embryos or tissues	NA

Powered by Editorial Manager® and ProduXion Manager® from Aries Systems Corporation

• Field research<br><br>Write "N/A" if the submission does not require an ethics statement.<br><br>General guidance is provided below. Consult the submission guidelines for detailed instructions. **Make sure that all information entered here is included in the Methods section of the manuscript.**	

Powered by Editorial Manager® and ProduXion Manager® from Aries Systems Corporation

**Format for specific study types**<br><br>**Human Subject Research (involving human participants and/or tissue)**<br>• Give the name of the institutional review board or ethics committee that approved the study<br>• Include the approval number and/or a statement indicating approval of this research<br>• Indicate the form of consent obtained (written/oral) or the reason that consent was not obtained (e.g. the data were analyzed anonymously)<br><br>**Animal Research (involving vertebrate animals, embryos or tissues)**<br>• Provide the name of the Institutional Animal Care and Use Committee (IACUC) or other relevant ethics board that reviewed the study protocol, and indicate whether they approved this research or granted a formal waiver of ethical approval<br>• Include an approval number if one was obtained<br>• If the study involved *non-human primates*, add *additional details* about animal welfare and steps taken to ameliorate suffering<br>• If anesthesia, euthanasia, or any kind of animal sacrifice is part of the study, include briefly which substances and/or methods were applied<br><br>**Field Research**<br><br>Include the following details if this study involves the collection of plant, animal, or other materials from a natural setting:<br>• Field permit number<br>• Name of the institution or relevant body that granted permission	
**Data Availability**<br><br>Authors are required to make all data underlying the findings described fully available, without restriction, and from the time of publication. PLOS allows rare exceptions to address legal and ethical concerns. See the PLOS Data Policy and FAQ for detailed information.	Yes - all data are fully available without restriction

A Data Availability Statement describing where the data can be found is required at submission. Your answers to this question constitute the Data Availability Statement and **will be published in the article**, if accepted.<br><br>**Important:** Stating 'data available on request from the author' is not sufficient. If your data are only available upon request, select 'No' for the first question and explain your exceptional situation in the text box.<br><br>Do the authors confirm that all data underlying the findings described in their manuscript are fully available without restriction?	
**Describe where the data may be found in full sentences. If you are copying our sample text, replace any instances of XXX with the appropriate details.**<br><br>• If the data are **held or will be held in a public repository**, include URLs, accession numbers or DOIs. If this information will only be available after acceptance, indicate this by ticking the box below. For example: *All XXX files are available from the XXX database (accession number(s) XXX, XXX.).*<br>• If the data are all contained **within the manuscript and/or Supporting Information files**, enter the following: *All relevant data are within the manuscript and its Supporting Information files.*<br>• If neither of these applies but you are able to provide **details of access elsewhere**, with or without limitations, please do so. For example:<br><br>*Data cannot be shared publicly because of [XXX]. Data are available from the XXX Institutional Data Access / Ethics Committee (contact via XXX) for researchers who meet the criteria for access to confidential data.*<br><br>*The data underlying the results presented in the study are available from (include the name of the third party*	CT models are available at morphosource

and contact information or URL).<br>• This text is appropriate if the data are owned by a third party and authors do not have permission to share the data.<br><br>* typeset	
Additional data availability information:	

Powered by Editorial Manager® and ProduXion Manager® from Aries Systems Corporation

Manuscript

Click here to access/download;Manuscript;Amphisbaenian inner ear manuscript.docx

Full title: The unique inner ear labyrinth of worm-lizards (Amphisbaenia: Squamata)

Short title: The innear ear of worm-lizards

Geneva E. Clark[1], Alessandro Palci[2], Rebecca J. Laver[3.4], Cristian Hernandez-Morales[5], Christian A. Perez-Martinez[6], Patrick J. Lewis[1], Monte L. Thies[1], Christopher J. Bell[7], Christy A. Hipsley[8], Johannes Müller[9], Ricardo Montero[10], Juan D. Daza[1]*

[1] Department of Biological Sciences, Sam Houston State University, Huntsville, TX 77341, USA.

[2] School of Biological Sciences, University of Adelaide, Adelaide, SA, Australia South Australian Museum, Adelaide, SA, Australia.

[3] Research School of Biology, Australian National University, Canberra, ACT, Australia.

[4] School of BioSciences, The University of Melbourne, Parkville, Victoria, Australia Museums Victoria, Melbourne, Victoria, Australia.

[5] Department of Biology, The University of Texas at Arlington, Arlington, TX, 76019, USA.

[6] Department of Biological Sciences, University of Missouri, Columbia, MO 65211, USA.

[7] Jackson School of Geosciences, The University of Texas at Austin, Austin, TX, USA.

[8] Department of Biology, University of Copenhagen, Copenhagen, Denmark.

[9] Museum für Naturkunde, Leibniz-Institut für Evolutions- und Biodiversitätsforschung, Invalidenstr. 43, D-10115 Berlin, Germany Berlin-Brandenburg Institute of Advanced Biodiversity Research – BBIB, Altensteinstr. 34, D-14195 Berlin, Germany

[10] Universidad Nacional de Tucumán, San Miguel de Tucumán, Tucumán, Argentina

*Telephone/email, corresponding author: +1 936-294-4250/ juand.daza@gmail.com

Abstract

Amphisbaenians are the most specialized group of reptiles for dwelling in fossorial habitats. Studies of the morphology of the inner ear based on ~~cast~~ derived from High-resolution computed tomography (HRCT) data ~~has~~ become a widely used method to study this part of the ear system. Previous researchers also have used these 3D models to make inferences about the ecology of fossil taxa. Although squamates have been widely studied using these methods, amphisbaenians have received relatively low attention. In this paper we survey the anatomy of the inner and middle ear in these fossorial reptiles using HRCT models. We describe qualitatively and quantitatively (using 3D morphometrics) the anatomy of the inner ear. Amphisbaenians are very diverse in skull anatomy, especially the configuration of the snout, which reflects the correlation with digging modes. We demonstrate that the ear also exhibits a large diversity of configurations, which we propose to be adaptations to the environments where they live, especially soil types. Results from these analyses also ~~allow to~~ describe 11 new phylogenetic characters that can be of some utility in future phylogenetic analyses.

Introduction

Members of the squamate clade Amphisbaenia are commonly known as worm lizards. They are a group of highly specialised lizards with elongated bodies, rudimentary eyes, and, except for one genus (*Bipes*), they are completely limbless [1-3]. These reptiles evolved for a subterranean mode of life and have developed one of the most curious locomotion modes among lizards – rectilinear locomotion – which is most often associated with snakes [4]. In relation to their subterranean habitats, amphisbaenians developed a diversity of adaptations that are reflected in their skeletal morphology [5], and also the loss of the eardrum [6].

Morphological descriptions of amphisbaenian skulls have mostly focused on head shape, particularly ~~in~~ modifications ~~on~~ the anterior portion [7-9], which is directly related to their subterranean lifestyle. The heads of amphisbaenians have diverse forms characterised by the snout, including rounded (e.g., most *Amphisbaena*, *Bipes*, *Blanus*, *Bronia*, *Cadea*, *Chirindia*, *Cynisca*, *Trogonophis*, *Loveridgea*, and *Zygaspis*), spade-headed (*Agamodon*, *Diplometopon*, *Pachycalamus*), keel-headed (*Ancylocranium*, *Anops*,

Baikia, *Geocalamus*, *Mesobaena*), and shovel-headed (*Aulura, Dalophia, Monopeltis, Leposternum*, and *Rhineura*). The diversity of head shapes reflects variation in the primary contact surface with the substrate and the associated methods of burrowing [2, 3, 5], and documents convergent evolution across the different clades [10, 11].

In contrast to the snout region, the posterior portion of the skull in fossorial groups remains poorly studied (i.e., the braincase and otic capsules), especially the sensorial structures including the hearing and equilibrium organs. This produces a conceptual gap in understanding the evolution of the squamate ear apparatus [12], which functions in hearing and balance and is strongly linked to phylogeny [13]. One example is variation of the columella configuration (e.g., innervation, size, and orientation; Figure 1), whose structure and anatomical detail are largely understudied.

Digital endocasts of the inner ear labyrinth (inner ear for simplicity) are now a widely used source of data [14-16] to address a diversity of biological questions, including the fossorial or aquatic origin of snakes [17, 18], and the correlations among shape, ecology, and locomotion in limb-reduced skinks [19]. Previous authors indicated that a distinct morphology is expressed in burrowers and aquatic forms, which therefore presupposes that amphisbaenians should express a similar morphology [18]. Previous comparative analyses of the middle ear described amphisbaenians as having variation in the columella and presence of the extracolumella [20, 21], which further suggests that differences might also extend into the inner ear.

Among squamates, ground vibrations are transmitted to the inner ear (osseous labyrinth), which is formed by three major components: the semicircular canals, the vestibule (sacculus), and the lagena. In this paper we follow mostly Weber's terminology [22], but we prefer the term lagena instead of cochlea [or endosseous cochlear duct 15] — cochlea is a more adequate term for the lagena of mammals, where this part of the inner ear is more spiral, analogous to a snail shell (coclea in latin). The two terms are often used interchangeable and refer to homologous structures [23].

Amphisbaenians have a typical fossorial inner ear, which is compact in appearance [24, 25] and is characterized by a large fenestra ovalis (consistent with the large stapedial footplate), a large spherical vestibule that occupies most of the space surrounded by the three narrow semicircular canals [6, 22, 24-26], and a lateral semicircular canal typically

in contact with (or closely approaching) the sacculus [18]. A constriction usually marks a separation between the sacculus and the lagena [15, 27].

We examined morphological variation in the inner ear of amphisbaenians, with the intent to corroborate whether there is a consistent inner ear morphology, or alternatively, variation across clades. Ideally, variation would be studied in the context of ecological guilds, but given the cryptic nature of these reptiles, their microhabitat preference remains largely unknown, and knowledge about their burrowing adaptations are restricted to snout shape and digging methods [28] which are convergent [3]. We drew from High-Resolution Computed Tomography (HRCT) data to generate endocasts of the labyrinth of the inner ear, and our approach included a combination of anatomical descriptions (qualitative comparison) and three-dimensional (3D) geometric morphometric (quantitative) comparisons to characterize morphological variation in the inner ear across amphisbaenians.

Materials and methods

1. HRCT data collection

We included 19 amphisbaenian species, representing all the major clades [According to 3, 29]. Specimens were CT scanned at three institutions: The University of Texas High-Resolution X-ray Computed Tomography Facility (UTCT) located at Austin, Texas, USA; Adelaide Microscopy Facility at The University of Adelaide, at Adelaide, SA, Australia; and the Natural History Museum of Berlin, Germany.

Most specimens were scanned at UTCT in ~~an~~ ZEISS Xradia MicroCT. *Amphisbaena vermicularis* was scanned using a Skyscan 1076 micro-CT. *Blanus cinereus* and *Cadea blanoides* were scanned using a GE Phoenix Nanotom S. The list of species and specimens is in Table 1.

2. Endocast rendering

Endocasts of the inner ears were rendered using the program Avizo Lite v9.5.0 (Thermo Fisher Scientific, 2018). The steps to create the endocast were as follows: 1) using the interpolate function and a rectangle in the selection tool, we created a box around the inner ear; 2) with this box selected, we created an inverse selection and added and locked this material, this creates a solid cast around the portion of the skull that bears the inner

ear; 3) all the bone contained inside the cast was selected, added, and locked in a second material; and 4) with this new selection we used the function selection to fill all slices. Areas from the endocast that were missing (due to differences in density) were added manually to complete the model. We used a diversity of selection tools, and cutting away portions of bone that might continue as part of the model. Images of the inner ear were obtained in dorsal, lateral, and medial views, using the whole skull as a guide for orientation. Due to variable head morphology in amphisbaenians, establishing a plane of orientation is extremely difficult, for this reason we standardized the lateral orientation maintaining the basicranium horizontally as indicated in Figure 2. From ~~the~~ position, we rolled counterclockwise the head 90 degrees to get the dorsal view and finally 180 degrees to get the ventral view.

3. Landmarking & geometric morphometric analysis

We quantified inner ear shape variation by placing 47 landmarks (seven fixed ~~an~~ 40 sliding semi-landmarks) on surface files (.ply format) of the ~~above~~ endocasts using the program Landmark Editor v3.6 [30], following a landmarking procedure previously developed for squamate reptiles [17]. To place amphisbaenians in the context of Squamata, we combined our new data with a previous squamate data set [17], for a total of 99 specimens, 58 snake, 21 amphisbaenian, and 20 other lizard species. The sample also includes 22 generalist, 15 arboreal, 6 aquatic, 11 semiaquatic, and 45 fossorial species, Supporting Information S2.

Landmark 48 (corresponding to the ventral end of the lagena) in the previous data set [17] was excluded, due to difficulty in establishing homology: in many amphisbaenians, the lagena is relatively small and does not protrude ventrally to the fenestra ovalis. Also, note that landmark 47 was placed approximately in the center of the vestibule (Figure 3C); however, when this was not the case (i.e., the vestibule was not hemispherical), the landmark was placed in the circumcenter by placing it at a point that corresponded to the center of the circumscribed circle best fitting the outline of the vestibule. Landmark configurations on the amphisbaenian inner ear endocast are shown in Figure 3. Measurement error for the landmarking scheme was found to be negligible [17], hence it is not discussed further ~~herein~~. The landmark configurations were scaled and aligned with a Procrustes superimposition using the package ‘geomorph’ v3.0.5 [31] in R v3.3.3 (R

Core Team, 2017; https://www.r-project.org/). Centroid size, defined as the square root of the sum of squared distances of each landmark to the centroid of the configuration, was used as an overall index of inner ear size. Principal Components Analysis (PCA) and statistical tests for correlation between centroid size and PC1 were performed using the same R package. Aligned coordinates used for our analysis are available in the Supporting Information S1.

4. Morphological comparisons and phylogenetic implications

Variation observed using qualitative and quantitative methods were expressed as transformation series and the character states were mapped using parsimony in Mesquite v. 3.81 [32] onto an existing total evidence amphisbaenian phylogeny [33]. That phylogeny was estimated using five genes and extensive phenotypic data [34]. For this paper, we propose these characters and explore their distribution, but they should be tested more rigorously in a phylogenetic analysis.

Results

Gans and Wever [26] described the anatomy of the ear and hearing mechanisms in Amphisbaenia and other reptiles more than 40 years ago [5, 6, 22]. Their model for the amphisbaenian ear can be interpreted as a three-component system: 1) a receptor, which in some amphisbaenians corresponds to the infralabial scales instead of the tympanic membrane; 2) a transmission segment, formed by two middle ear elements, the extracolumella and columella; and 3) a sound and equilibrium processing system, formed by structures located in the inner ear. Below we describe the middle and inner ear using two approaches, qualitatively using a character-based approach. We also quantify the shape of the inner ear using 3D morphometrics. The variation captures with these approaches, was mapped onto a phylogeny.

1. Middle ear transmission segment:

Not all amphisbaenians have an elongated extracolumella, and that element does not develop the genus *Bipes* at the base of the clade Amphisbaeniformes. The columella fits in the fenestra ovalis in four different orientations (Figure 1). When the extracolumella is elongated, it runs anteriorly and parallel to the mandibular ramus. In the two more basal

families (Rhineuridae and Bipedidae) this element differs considerably. In *Rhineura*, the extracolumella is directed forward, but instead of being long and slender, it is short and flattened (paddle-like). In the remaining species studied, the extracolumella is long and slender, and its length appears to be affected by body size. For example, in the sampled specimens of the genus *Zygaspis*, skull length ranged from 5.96 mm (*Zygaspis quadrifrons*) to 10.40 mm (*Zygaspis nigra*). The extracolumella also exhibits considerably morphological variation, including, spoon-shaped, ball shaped, or linear [35]. Within the size range of *Zygaspis*, the extracolumella becomes proportionally smaller and stouter as the specimens increase in size. This trend is also observed in the extracolumella of other Amphisbaeniformes (i.e. all amphisbanians exclusive of Bipediade), in which small individuals have a longer, ~~slenderer~~ extracolumella, and in larger individuals it is proportionally short and thick.

The columella in general has a short shaft and broad footplate. There is high variation in the shape of the columella from the shaft being directed laterally and contacting the extracolumella almost at a right angle (e.g., *Rhineura*), to a columella with a shaft abutting the extracolumella anteriorly (e.g., *Zygaspis*). The differences in the columella translate into different orientations of the fenestra ovalis, which can be seen in the endocasts, where the fenestra ovalis displays varying orientations when seen in lateral view with the floor of the braincase horizontally oriented. (Figure 4). Although this rotation spans an almost continuous spectrum across species, three broad categories can be identified: 1) columella footplate facing medially (*Rhineuridae*), 2) columella footplate facing dorsally (*Bipedidae*), 3) columella footplate facing ventromedially (Blanidae, Cadeidae, Trogonophidae, amphisbaenid *Geocalamus*), and 4) columella footplate facing posteriorly (amphisbaenids *Zygaspis* and *Amphisbaena*).

2. Inner ear sound and equilibrium processing system:

Semicircular canals: Not all amphisbaenians exhibit the extreme compact shape of the osseous labyrinth previously described for fossorial forms [18]. Although the semicircular canals tend to be within close proximity of the vestibule in many species, some amphisbaenian species fail to develop the distinctive compact appearance reported in other groups such uropeltid snakes [25], fossil snakes such as *Dinilysia patagonica*, and burrowing snakes such as *Xenopeltis unicolor* [18, 36]. In some species, there is a

gap between the anterior and posterior semicircular canals and the vestibule (Fig 3). This spacing was observed in *Amphisbaena*, *Bipes*, *Cadea blanoides*, *Rhineura floridana*, and two species of *Zygaspis* (*Z. ferox* and *Z. nigra*). Another feature that characterizes other fossorial squamates is that the lateral semicircular canal is in contact with the sacculus [17, 18, 25, 27, 36]. This trait is ~~only present in~~ *Rhineura floridana* and *Trogonophis wiegmanni* (Fig 3K–L), and to some extent in *Bipes*, while in the other amphisbaenians, the lateral semicircular canal is shifted slightly ventrally and positioned closer to the constriction that marks the junction between the vestibule and the lagena (Fig 4). The lateral semicircular canal is anterodorsally oriented in *Rhineura floridana* (Figure 4A) and to a lesser extent in Bipedidae (especially in in *B. canaliculatus*, Figure 4C). In *R. floridana*, the angle of orientation of the lateral semicircular canal matches the inclination of the snout, a pattern also observed in HRCT models of the fossil *Rhineura hatcherii* [37].

Another trait associated with other fossorial squamates is thinning of the semicircular canals. This trait is not present in all the amphisbaenians studied and was most notable only in *Cadea* and *Diplometopon* (Figure 4E, F). The appearance of the semicircular canals is indeed thick, independent of the size of the specimens, for example in large-sized species (e.g., *Amphisbaena alba*, *Rhineura*, *Trogonophis*) or in very small ones (*Zygaspis kafuensis*, *Z. vandami*, *Z. quadrifrons*). In the latter, the relatively large diameter of the semicircular canal can be attributed to a functional constraint upon these structures.

In *Rhineura*, the anterior ampulla and anterior semicircular canal are arranged forming almost a 90-degree angle. This is unique compared with other amphisbaenians which have a more curved junction that does not form a right angle. In Cadeidae and Trogonophidae, the anterior ampullae are reduced. The anterior semicircular canal in *A. alba* is the most widely separated from the vestibule among those species studied; it projects forward before curling back around to meet the anterior ampulla (Figure 4J). There is a similar pattern in *A. fuliginosa* (Figure 4K), although not as marked. In the species of *Zygaspis* examined, the anterior semicircular canal is at the same level as the anterior ampulla (Figure 4H).

Vestibule and lagena: We observed remarkable variation in inner ear structure across amphisbaenians, suggesting that size, mode of burrowing, and dwelling substrate might affect how these animals hear and process information about their position in space. In all specimens, the vestibule portion is proportionally larger than the lagena and in some forms, the volume of the vestibule is approximately eight times the size of the lagena (e.g., *Bipes canaliculatus*, *Diplometopon*, and *Trogonophis*), compared to about 4–5 times the size of the lagena in other species. Considering members of the genus *Zygaspis*, these proportions are affected by overall body size: in the largest form (*Zygaspis nigra*), the vestible is about five times the size of the lagena, while in the smallest forms (*Zygaspis vandami*, *Z. kafuensis*), the vestible is about three times the size of the lagena. As observed in previous studies [25, 36], in other burrowing squamates, the vestibule is nearly spherical, and this is something observed in all species sampled, although with various degrees of distortion. We also observed a well-marked ridge in the lateral surface of *Bipes* and *Blanus* (Figure 4B–D).

Among amphisbaenians, the perilymphatic duct was found attached to the lagena in all species except *Rhineura floridana* (Figure 4A) and *Zygaspis kafuensis* (Figure 4H), where it was a structure separate from the ECD. The posterior end of the perilymphatic duct in some forms develops a loop (e.g., *Zygaspis kafuensis* Figure 4H; and *Geochalamus acutus* Figure 4I).

Rhineura floridana differs from other amphisbaenians in the way that the anterior and posterior utriculi bound the dorsal border of the acoustic recess. In that species, the dorsal border of the acoustic recess is horizontal, while in the other species, it is deeply notched.

3. Geometric morphometric analysis:

The first three principal components account for 64% of the variance in shape of the inner ear endocast (Figure 5). In the plot of PC1 vs PC2, the inner ears of amphisbaenians are clearly set apart from those of all other squamates, as they all have values of PC1 below -0.1. The only squamates that fall closer to the amphisbaenian inner ear morphospace are the scincid *Acontias meleagris* (#2 in Figure 5), the anniellid *Anniella pulchra* (#10), the uropeltid snake *Teretrurus sanguineus* (#69), and the pipe-snake *Cylindrophis ruffus* (#25). Interestingly, all of these forms share a fossorial ecology with amphisbaenians. In the plot of PC2 vs PC3, there are no clear distinctions for

amphisbaenians, which fall around the center of the shape distribution, but with a narrow spread across PC3 and a much broader spread across PC2.

Negative values of PC1 mostly correspond to an almost circular configuration of the semicircular canals, especially on the lateral axis: this can be explained by a considerably inflated vestibule. Positive values, on the other hand, mark a pinching of the lateral profile of the anterior and posterior semicircular canals towards the baricentrum of the inner ear. This lateral profile is due to a change in orientation of the plane of the semicircular canals, which lie closer to the horizontal plane. Positive values of PC1 are also associated with a relatively larger lateral ampulla, and a relatively smaller vestibule.

Negative values of PC2 correspond to changes from a globular (positive values) to a depressed (negative values) vestibule. Changes in the vestibule are correlated with an expansion of the semicircular canals. Consequently, the anterior and posterior semicircular canals in forms with highest values for PC2 appear shifted downward. Moreover, positive values for PC2 also mark a shift of the vestibule towards the posterior end (especially notable in dorsal view in Figure 5), and an anterior semicircular canal that is mediolaterally broader.

Negative values for PC3 capture the expansion or contraction of the lateral ampulla relative to the rest of the vestibule, and a posterior shift of the vestibule. Positive values of PC3 also mark a shorter anterior semicircular canal.

Finally, we tested for a correlation between inner ear size (approximated by centroid size) and shape variation (approximated by PC1) [38] and found that there is no significant correlation between the two, regardless of the statistical test used (Pearson: t_{17}=-2.0792, cor = -0.4503, p-value=0.05305; Kendall: T=65, tau=-0.2398, p-value=0.1637; Spearman: S=1564, rho=-0.3719, p-value=0.1176).

4. Character mapping:

Based on the observed variation and morphometric characters, 11 new characters are proposed and consequently mapped onto the existing phylogeny of amphisbaenians (Figure 6).

1. Fenestra ovalis: (0) facing laterally (Figure 1, e.g. *Diplometopon*), (1) facing ventrally (Figure 1, e.g. *Bipes)*, (2) facing anterolaterally (Figure 1, e.g. *Rhineura*), (3) facing anteriorly (Figure 1, e.g. *Zygaspis*).

2. Vestibule: (0) not globular, (1) globular (all amphisbaenians, Figures 2, 4).
3. Vestibule: with lateral ridge: (0) absent (Figure 7A), (1) present (figure 7B).
4. SSC: (0) not arranged in a circular trajectory, (1) arranged in a circular trajectory (all amphisbaenians, Figure 4).
5. SSC: (0) separated from the vestibule (Figure 7A), (1) close to the vestibule (Figure 7B).
6. Lateral SSC: (0) at the vestibule-lagena junction (Figure 2E), (1) wrapping around the vestibule (Figure 3B).
7. Lateral SSC: (0) mainly in the horizontal plane (Figure 3B), (1) anterodorsally oriented (Figure 2E).
8. Lagena: (0) large with a small portion representing the fenestra ovalis (and columella footplate), (1) reduced, limited mostly to the size of the fenestra ovalis (and columella footplate, all amphisbaenians, Figure 4).
9. Perilymphatic duct: (0) attached to the lagena (Figure 4B), (1) separated from lagena (Figure 2F).
10. Posterior end of perilymphatic duct: (0) without a loop (Figure 7B), (1) forming a loop (Figure 7A).
11. Anterior and posterior utriculus: (0) bordering the acoustic recess, dorsal border mostly horizontal (Figure 2F), (1) acoustic recess dorsal border deeply notched (Figure 4).

Discussion

Despite the fact that amphisbaenians are obligate burrowers and represent the most specialized fossorial group of squamates [17], their inner ear is not uniform. Analysis of inner ear endocasts of a broad group of amphisbaenians illustrates considerable morphological diversity and presents a new source of characters that have utility in future phylogenetic analyses.

Previously, the compacted shape of the inner ear was used as an argument supporting the fossorial ecology of the fossil snake *Dinilysia patagonica* [18], and considering its debated nested position within the crown group Ophidia [39, 40], it was used to support the argument for the burrowing origin of snakes [18]. This interpretation has been disputed, given that that morphology is also present in semi-fossorial and semi-

aquatic forms [17]. Previous workers ~~has~~ also demonstrated that many dedicated burrowing snakes have large spherical vestibules [36], although that does not seem to be the case in obligate burrowing blind snakes (Scolecophidia).

The presence of a large footplate in the columella and thin semicircular canals wrapped tightly to the vestibule are two other traits commonly associated with fossoriality. Amphisbaenians have a large stapedial footplate. In fact, the fenestra ovalis area is the most prominent feature ~~of the~~ of the lagena. A globular and enlarged vestibule previously was proposed to be associated with fossoriality [18]. In this study, we found that amphisbaenians follow this pattern; however, they show differences that might be correlated with their dissimilar middle ear. The amphisbaenians with the most expanded vestibule include Bipedidae, Blanidae, Cadeidae and *Diplometopon*. Previously we stated that amphisbaenians inhabit localities with different soil types, with same species being specialized for particular soil types, and others inhabiting various different soil types [41, 42], making ecological associations to specific burrow properties difficult. However, we note that species with the proportionally largest vestibule dwell mainly in sandy soils [43], suggesting that this type of habitat might offer less restriction to movement (and as a consequence is associated with expansion of the otic capsules) than more compacted or rocky soils. One exception is *Rhineura*, which is also found in sandy woodlands or sandhills [44].

Given their cryptic nature, habitat preferences and tolerances of living amphisbaenians are difficult to accurately describe. Future studies should focus on studying the behaviour of amphisbaenians beyond descriptions of digging style (e.g. soil preference, microhabitat, biomechanics), this information can be extrapolated to fossil forms, based on their inner ear structure. These data could be valuable in drawing stronger correlations with morphological variation of the inner ear, because both the locomotion of amphisbaenians and transmission of ground-borne vibrations vary according to sediment type.

Amphisbaenians show striking convergence with limbless amphibian caecilians [45], another obligate burrowing vertebrate (except typhlonectids, which are aquatic caecilians), in some of the middle and inner ear structures. In both groups, the stapedial footplate and fenestra ovalis are very large. The vestibule is also globular and enlarged,

which indicates parallel adaptations to perceiving ground-borne vibrations. However, one major difference is that the semicircular canals in caecilians are thick and oriented in a different position, being mostly dorsal to the vestibule rather than surrounding it. The inner ears of caecilians have a totally different configuration; however, their shared similarities with amphisbaenians presupposes the ear of these two groups function in a similar way.

This is a prime example that provides new information about the diversity ~~on~~ inner ear morphology in the most specialized fossorial squamates and might help to improve our knowledge ~~on~~ fossorial ecologies. At the same time our data suggest that comparisons among groups with similar ecology need to be done more carefully, given the unique combination of characters (morphological and morphometric) associated with fossoriality.

Acknowledgements

We thank Jessie Maisano and Matt Colbert from the University of Texas – High-Resolution X-Ray CT Facility for scanning most of the specimens used in this study. This project started as an independent undergraduate research project from GEC in 2017 at Sam Houston State University, and gradually ~~growth~~ into a larger survey. Funding was received from multiple agencies, but we want to specially thank The Carl Gans Fund for sponsoring GEC to present part of this work in the 2016 meeting of the American Society of Ichthyology & Herpetology.

Author Contributions

Conceptualization: Christopher J. Bell, Geneva E. Clark, Juan D. Daza, Alessandro Palci, Patrick J. Lewis, Monte L. Thies.

Methodology: Geneva E. Clark, Juan D. Daza, Rebecca J. Laver, Cristian Hernández-Morales, Christian A. Perez-Martinez, Alessandro Palci.

Writing – original draft: All authors gathered data and helped draft the manuscript.

Writing – review & editing:

Funding: The Carl Gans Fund, Wilson-Warner, James D. Long and the Biology Department Scholarships supported Geneva E. Clark. Sam Houston State University Enhancement Research Grant (2014) to Juan D. Daza, Patrick J. Lewis, Monte L. Thies supported obtaining data for this study and funds from the Jackson School of Geosciences at The University of Texas at Austin to Christopher J. Bell.

References

1. Pianka ER, Vitt LJ. Lizards: Windows to the Evolution of Diversity. 1st ed. Berkeley, Los Angeles, & London: University of California Press; 2003. 346 p.
2. Gans C. The characteristics and affinities of the Amphisbaenia. Trans Zool Soc London. 1978;34:347–416. doi: 10.1111/j.1096-3642.1978.tb00376.x
3. Kearney M. Systematics of the Amphisbaenia (Lepidosauria: Squamata) based on morphological evidence from recent and fossil forms. Herpetol Monogr. 2003;17:1–74. doi: 10.1655/0733-1347(2003)017[0001:SOTALB]2.0.CO;2.

4. Hohl LSL, Loguercio MFC, Buendía RA, Almeida-Santos M, Viana LA, Barros-Filho JD, et al. Fossorial gait patterns and performance of a shovel-headed amphisbaenian. J Zool. 2014;294:234–40. doi: 10.1111/jzo.12173.

5. Gans C. Biomechanics: An approach to vertebrate biology. Philadelphia: J.B. Lippincott Company; 1974. 261 p.

6. Wever EG, Gans C. The ear in Amphisbaenia (Reptilia); further anatomical observations. J Zool. 1973;171:189–206. doi: 10.1111/j.1469-7998.1973.tb02215.x.

7. Ribeiro S, Castro-Mello C, Nogueira C. New Species of *Anops* Bell, 1833 (Squamata, Amphisbaenia) from Jalapão Region in the Brazilian Cerrado. J Herpetol. 2009;43:21–8. doi: 10.1670/07-299R1.1

8. Vanzolini PE. On *Anops* (Reptilia: Amphisbaenia: Amphisbaenidae). Pap Avulsos Zool. 1999;41:1–37.

9. Gans C. Studies on amphisbaenids (Amphisbaenia, Reptilia) 1. A taxonomic revision of the Trogonophinae, and a functional interpretation of the amphisbaenid adaptive pattern. Bull Amer Mus Nat Hist. 1960;119(3):129–204.

10. Kearney M. Systematics of the Amphisbaenia (Lepidosauria: Squamata) based in morphological evidence from recent and fossil forms. Herpetol Monograph. 2003;17:1–74.

11. Montero R, Gans C. An atlas of amphisbaenian skull anatomy. In: Gans C, Gaunt AS, Adler K, editors. Biology of the Reptilia, Volume 21, Morphology I (The Skull and appendicular locomotor apparatus of Lepidosauria). Ithaca, USA: Society for the Study of Amphibians and Reptiles; 2008. p. 621–738.

12. Lombard RE, Bolt JR. Evolution of the tetrapod ear: an analysis and reinterpretation. Biological Journal of the Linnean Society. 1979;11:19–76. doi: 10.1111/j.1095-8312.1979.tb00027.x.

13. Palci A, Hutchinson MN, Caldwell MW, Lee MSY. The morphology of the inner ear of squamate reptiles and its bearing on the origin of snakes. R Soc Open Sci. 2017;4:170685. doi: 10.1098/rsos.170685.

14. Balanoff AM, Bever GS, Colbert MW, Clarke JA, Field DJ, Gignac PM, et al. Best practices for digitally constructing endocranial casts: examples from birds and their dinosaurian relatives. J Anat 2016;229:173–90. doi: 10.1111/joa.12378.

15. Witmer LM, Ridgely RC, Dufeau DL, Semones MC. Using CT to peer into the past: 3D visualization of the brain and ear regions of birds, crocodiles, and nonavian dinosaurs. In: Endo H, Frey R, editors. Anatomical Imaging: Towards a New Morphology. Tokyo: Springer; 2008. p. 67–87.

16. Olori JC. Digital endocasts of the cranial cavity and osseus labyrinth of the burrowing snake *Uropeltis woodmansoni* (Alethinophidia: Uropeltidae). Copeia. 2010;2010:14–26. doi: 10.1643/CH-09-082

17. Palci A, Hutchinson MN, Caldwell MW, Lee MSY. The morphology of the inner ear of squamate reptiles and its bearing on the origin of snakes. Roy Soc Open Sci 2017;4:170685. doi: 10.1098/rsos.170685.

18. Yi H, Norell MA. The burrowing origin of modern snakes. Sci Adv. 2015;1:e1500743. doi: 10.1126/sciadv.15007

19. Camaiti M, Wiles J, Hutchinson MN, Hipsley CA, Chapple DG, Evans AR. Ecomorphological correlates of inner ear shape in Australian limb-reduced skinks (Scincidae: Sphenomorphini). Zool. J. Linn. Soc. 2023;199:994–1012. doi: 10.1093/zoolinnean/zlad074

20. Gans C, Wever EG. The Amphisbaenian Ear: *Blanus cinereus* and *Diplometopon zarudnyi*. Proc Nat Acad Sci. 1975;72:1487–1490. doi: 10.1073/pnas.72.4.1487

21. Sánchez-Martínez PM, Daza JD, Hoyos JM. Comparative anatomy of the middle ear in some lizard species with comments on the evolutionary changes within Squamata. Peerj. 2021;9(e11722). doi: 10.7717/peerj.11722

22. Wever EG. The Reptile Ear: Its Structure and Function. Princeton, New Jersey: Princeton University Press; 1978. 1024 p.

23. Kardong K. Vertebrates: Comparative Anatomy, Function, Evolution, 8th Edition. New York: McGraw-Hill 2019. 816 p.

24. Comeaux RS, Olori JC, Bell CJ. Cranial osteology and preliminary phylogenetic assessment of *Plectrurus aureus* Beddome, 1880 (Squamata: Serpentes: Uropeltidae). Zoological Journal of the Linnean Society. 2010;160(1):118–38. doi: 10.1111/j.1096-3642.2009.00595.x.

25. Olori JC. Digital endocasts of the cranial cavity and osseous labyrinth of the burrowing snake *Uropeltis woodmasoni* (Alethinophidia: Uropeltidae). Copeia. 2010;2010(1):14–26. doi: 10.1643/CH-09-082.

26. Gans C, Wever EG. The ear and hearing in Amphisbaenia (Reptilia). J Exp Zool 1972;179:17–34. doi: 10.1002/jez.1401790103

27. Daza JD, Bauer AM. Cranial anatomy of the pygopodid lizard *Aprasia repens*, a gekkotan masquerading as a scolecophidian. In: Emonds ORP, Powell GL, Jamniczky HA, Bauer AM, Theordor J, editors. All Animals Are Interesting: A Festschrift in Honour of Anthony P Russell. Germany: BIS-Verlag der Carl von Ossietzky Universität; 2015. p. 303–50.

28. Gans C. Biomechanics: An approach to vertebrate biology. Ann Arbor: University of Michigan Press; 1980. 272 p.

29. Vidal N, Azvolinsky A, Cruaud C, Hedges SB. Origin of tropical American burrowing reptiles by transatlantic rafting. Biol Lett. 2008;4(1):115-8. doi: 10.1098/rsbl.2007.0531

30. Wiley DF, Amenta N, Alcantara DA, Ghosh D, Kil YJ, Delson E. Landmark editor version 3.6: Institute for Data Analysis and Visualization. 3.6 ed. Davis: University of California; 2007.

31. Adams DC, Collyer ML, Kaliontzopoulou A, Sherratt E. Geomorph: Software for geometric morphometric analyses. R package version 3.0.5. https://cran.r-project.org/package=geomorph)2017.

32. Maddison WP, Maddison DR. Mesquite: a modular system for evolutionary analysis. Version 3.81 http://www.mesquiteproject.org. 2023.

33. Longrich NR, Vinther J, Pyron RA, Pisani D, Gauthier JA. Biogeography of worm lizards (Amphisbaenia) driven by end-Cretaceous mass extinction. Proc Biol Sci. 2015;282:20143034. oi: 10.1098/rspb.2014.3034.

34. Gauthier J, Kearney M, Maisano JA, Rieppel O, Behlke A. Assembling the squamate tree of life: perspectives from the phenotype and the fossil record. Bull Peabody Mus Nat Hist. 2012;53:3-308

35. Meza A, Bell CJ, Daza JD, Thies ML, Lewis PJ. Variation in the cranial osteology of the amphisbaenian genus *Zygaspis* based on high-resolution x-ray computed tomography. Anat Rec. 2024; 307:475–494. doi: 10.1002/ar.25321.

36. Yi H. Using adaptive traits in the ear to estimate ecology of eary squamates. In: Gower DJ, Zaher H, editors. The origin and early evolutionary history of snakes: Cambridge University Press, Padstow Cornwall; 2022. p. 271–93.

37. Kearney M, Maisano JA, Rowe T. Cranial anatomy of the extinct amphisbaenian *Rhineura hatcherii* (Squamata, Amphisbaenia) based on high-resolution X-ray computed tomography. J Morphol. 2005;264:1–33. doi: 10.1002/jmor.10210

38. Hollander M, Wolfe DA, Chicken E. Nonparametric statistical methods. 3rd ed. Hoboken, New Jersey: John Wiley & Sons, Inc.; 2013. 848 p.

39. Zaher H, Mohabey DM, Grazziotin FG, Wilson Mantilla JA. The skull of *Sanajeh indicus*, a Cretaceous snake with an upper temporal bar, and the origin of ophidian wide-gaped feeding. Zool J Linn Soc. 2023;197:656–97. doi: 10.1093/zoolinnean/zlac001

40. Zaher H, Scanferla CA. The skull of the Upper Cretaceous snake *Dinilysia patagonica* Smith-Woodward, 1901, and its phylogenetic position revisited. Zool J Linn Soc. 2012;164(1):194–238. doi s:10.1111/j.1096-3642.2011.00755.x

41. Kazi S, Hipsley CA. Conserved evolution of skull shape in Caribbean head-first burrowing worm lizards (Squamata: Amphisbaenia). Biol J Linn Soc. 2018;125:14–29. doi: 10.1093/BIOLINNEAN/BLY086

42. Hipsley CA, Müller J. Relict endemism of extant Rhineuridae (Amphisbaenia): testing for phylogenetic niche conservatism in the fossil record. Anat Rec. 2014;297:473–81. doi: 10.1002/ar.22853

43. Martín J, López P, Salvador A. Microhabitat selection of the amphisbaenian *Blanus cinereus*. Copeia. 1991;1991(4):1142–6.

44. Bartlett RD, Bartlett PP. A field guide to Florida reptiles and amphibians. Houston: Gulf Publishing Company; 1999. xvi+280 p.

45. Maddin HC, Sherratt E. Influence of fossoriality on inner ear morphology: insights from caecilian amphibians. J Anat. 2014;225:83–93. doi: 10.1111/joa.12190

Supportive Information

S1 Aligned coordinates used in the 3D morphometric analyses.

S2 List of species and habitat preference of specimens used in the 3D morphometric analyses.

Figure Captions

Figure 1. HRCT scans showing variation in the morphology and position of the columella (blue) in the fenestra ovalis of four amphisbaenians, with skulls in left lateral and ventral views. Isolated columella in ventral view (bottom row), showing the orientation of the columella shaft: *Bipes biporus* (ventrally), *Diplometopon zarudnyi* (ventrolaterally), *Rhineura floridiana* (lanterolaterally), and *Zygaspis quadrifrons* (anteriorly).

Figure 2. Skull of *Rhineura floridana* (FMNH 31774) showing the position of the inner ear endocast in dorsal (A), ventral (B), lateral (C), and midsagittal (interior) (D) views. Detailed views of the endocast of the same specimen with parts labelled, in lateral (E), medial (F), dorsal (G), and ventral (H) views.

Figure 3. Position of landmarks (yellow) and semilandmarks (green) on the inner ear endocast of *Cadea blanoides* (ZMB 4082). Numbers indicate point landmarks on the X (A), medial (B) lateral, and (C) dorsal views.

Figure 4. Variation of inner ear labyrinths (right endocast) in representatives from six families of Amphisbaenians, with endocast color corresponding to family in the simplified phylogeny [33]. Each endocast is shown in three views, from top to bottom: lateral (anterior end to the right), medial (anterior end to the left) and dorsal (anterior end to the right). A) *Rhineura floridana* (FMNH 31774), B) *Bipes biporus* (CAS 126478). C) *Bipes canaliculatus* (CAS 134753), D) *Blanus cinereus* (ZMB 29178), E) *Cadea blanoides* (ZMB 4082), F) *Diplometopon zarudnyi* (FMNH 64429), G) *Trogonophis wiegmanni elegans* (FMNH 109462), H) *Zygaspis kafuensis* (NMZB-UM 30040), I) *Geocalamus acutus* (FMNH 62014), J) *Amphisbaena alba* (FMNH 195924), and K) *Amphisbaena fuliginosa* (FMNH 22847). 3D models illustrated here are available in

MorphoSource (Table 1). Scale bar in the endocasts equals 1 mm. Below, diceCT specimen of *Zygaspis kafuensis*, used to render the brain, inner ear endocast, columella and extracolumella. Live animal, *Amphisbaena schmidti* from Arecibo, Puerto Rico.

Figure 5. Morphospace distribution of 99 inner ear endocasts of squamate reptiles defined by the first three principal components (ordinary PCA). Projections of the Procrustes landmark configurations corresponding to the positive and negative extremes of each axis are also shown. The projections are in lateral (left or top) and dorsal (right or bottom) views with anterior to the right. The distribution of the amphisbaenians is highlighted by a 90% equal frequency ellipse (in pink). For a complete list of species names that correspond to the numbers and abbreviations used in the plots see Supporting Information S2.

Figure 6. Characters proposed mapped onto a symplified phylogeny of amphisbaenians [33]. Character descriptions are provided in the text.

Figure 7. Endocasts of the inner ear of some amphisbaenians illustrating character states proposed in this paper. A) *Geocalamus acutus*, B) *Bipes canaliculatus*, C) *Amphisbaena alba*, D) *Amphisbaena fuliginosa*.

Family	Species	Specimen ID	MorphoSource ID	DigiMorph ID	Collection Locality
Rhineuridae	*Rhineura floridana*	FMNH 31774	594371	31774	Lakeland, Polk County, Florida, USA
Bipedidae	*Bipes biporus*	CAS 126478	594346	126478	La Paz, Baja California Sur, Mexico
Bipedidae	*Bipes canaliculatus*	CAS 134753	594351	134753	Rio Balsas, Guerrero, Mexico
Blanidae	*Blanus cinereus*	ZMB 29178	594356	–	Valladolid, Castilla y León, Spain
Cadeidae	*Cadea blanoides*	ZMB 4082	594361	–	Cuba
Trogonophidae	*Diplometopon zarudnyi*	FMNH 64429	594366	64429	Qatif Oasis, Saudi Arabia
Trogonophidae	*Trogonohis wiegmanni elegans*	FMNH 109462	–	109462	Morocco
Amphisbaenidae	*Amphisbaena alba*	FMNH 195924	594330	195924	Serranía de Santiago Chiquitos Prov., Santa Cruz, Bolivia
Amphisbaenidae	*Amphisbaena caeca*		–	–	Puerto Rico
Amphisbaenidae	*Amphisbaena fuliginosa*	FMNH 22847	594335	22847	Canal Zone, Panama
Amphisbaenidae	*Amphisbaena vermicularis*		–	–	
Amphisbaenidae	*Geocalamus acutus*	FMNH 262014	594341	262014	Dodoma, Tanzania
Amphisbaenidae	*Zygaspis dolichomenta*	R-147	–	147B	
Amphisbaenidae	*Zygaspis dolichomenta*	R15907	–	15907B	
Amphisbaenidae	*Zygaspis ferox*	MCZ R-182217	–	182217A	Silverstreams, Chimanimani Distr., Zimbabwe
Amphisbaenidae	*Zygaspis kafuensis*	NMZB-UM 30040	594377	30040A	
Amphisbaenidae	*Zygaspis nigra*	FMNH 133021	–	133021B	Barotseland
Amphisbaenidae	*Zygaspis quadrifrons*	FMNH 17751	–	17751B	Botswana
Amphisbaenidae	*Zygaspis quadrifrons*	TNHC 85060	–	85060A	Koanaka Hills, Ngamiland, Botswana
Amphisbaenidae	*Zygaspis vandami arenicola*	FMNH 268569	–	268569A	Zimbabwe
Amphisbaenidae	*Zygaspis violacea*	FMNH 265728	–	265728A	Zululand, KwaZulu-Natal, South Africa

Table 1. Specimens used in geometric morphometric analyses. CAS, California Academy of Sciences; FMNH, Field Museum of Natural History, Chicago; MCZ, Museum of Comparative Zoology, Harvard; MNMZB-UB, Natural History Museum of Zimbabwe, Bulawayo (formerly UM = Umtali Museum); TNHC, Texas Natural History Collections. MorphoSource and DigiMorph repository IDs are given, when available.

Figure 1

Click here to access/download;Figure;Figure 1 New.png

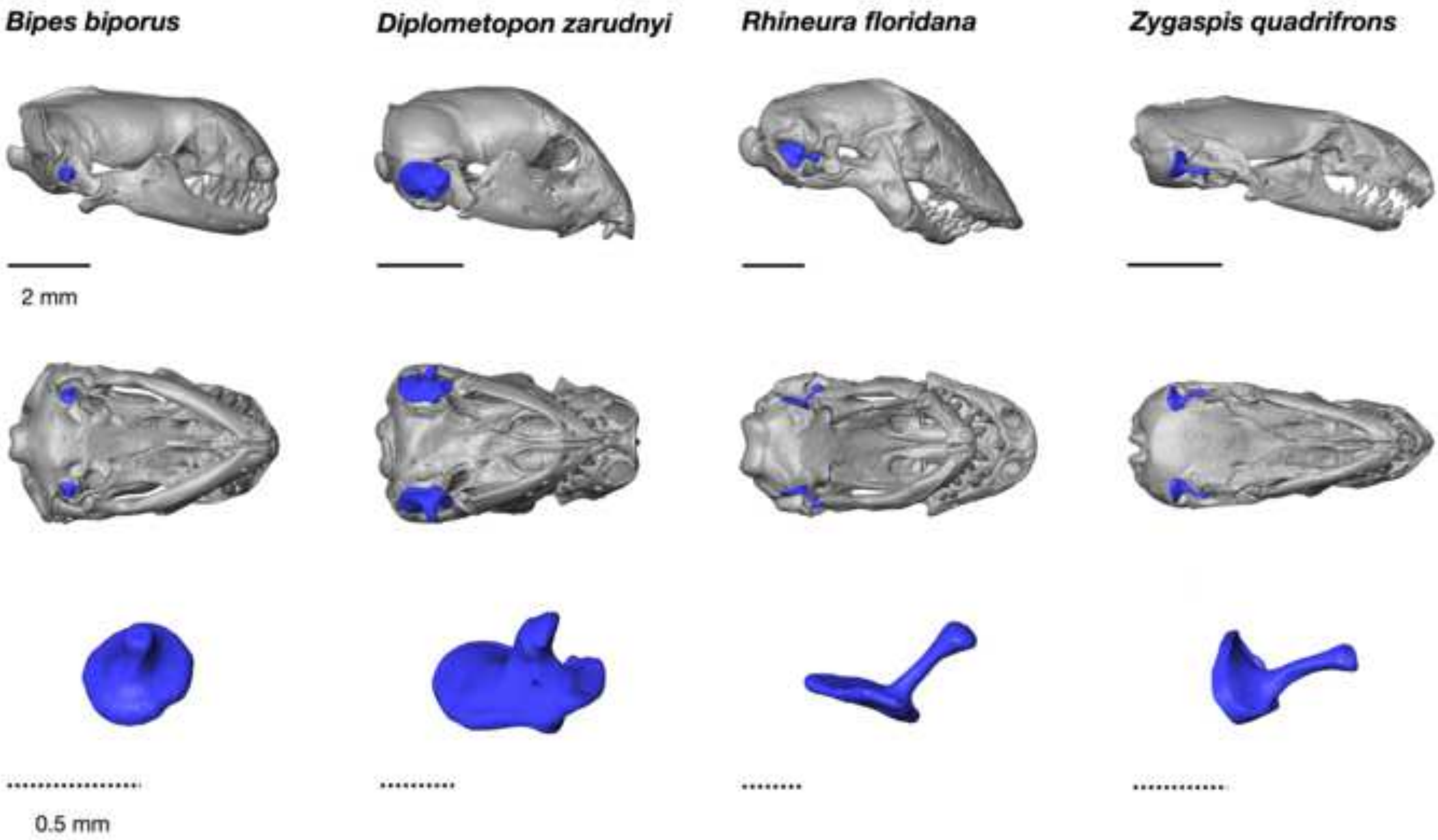

Figure 2

Click here to access/download;Figure;Figure 2.png

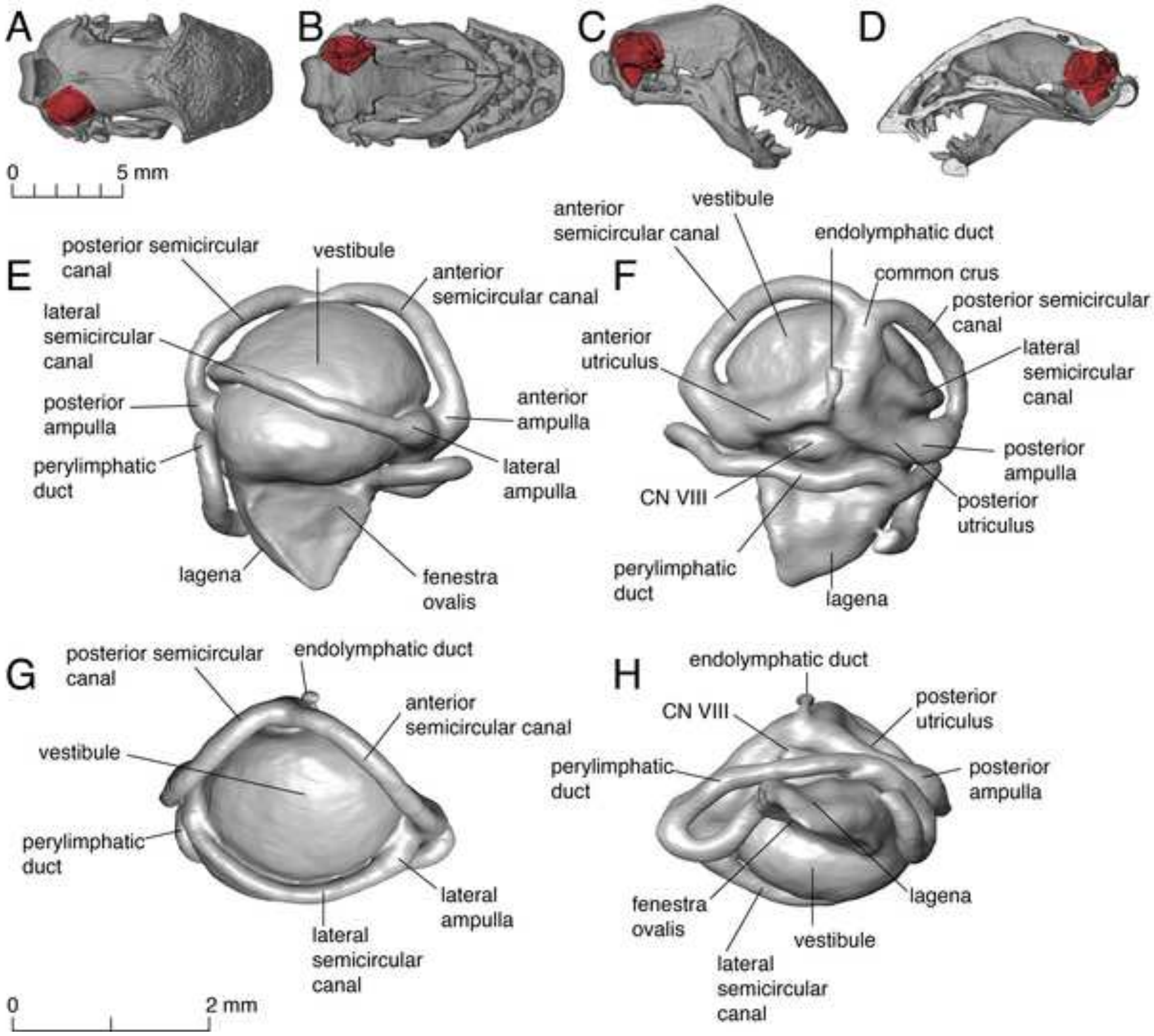

Figure 3 Click here to access/download;Figure;Figure 3.png

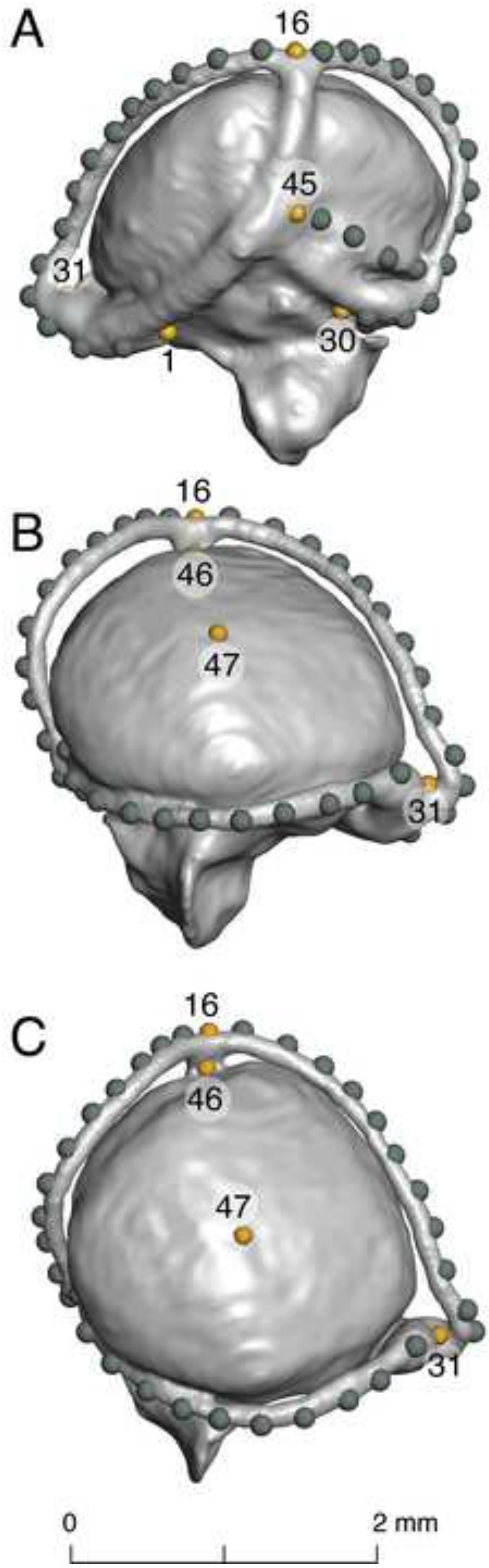

Figure 4

Click here to access/download;Figure;Figure 4.png

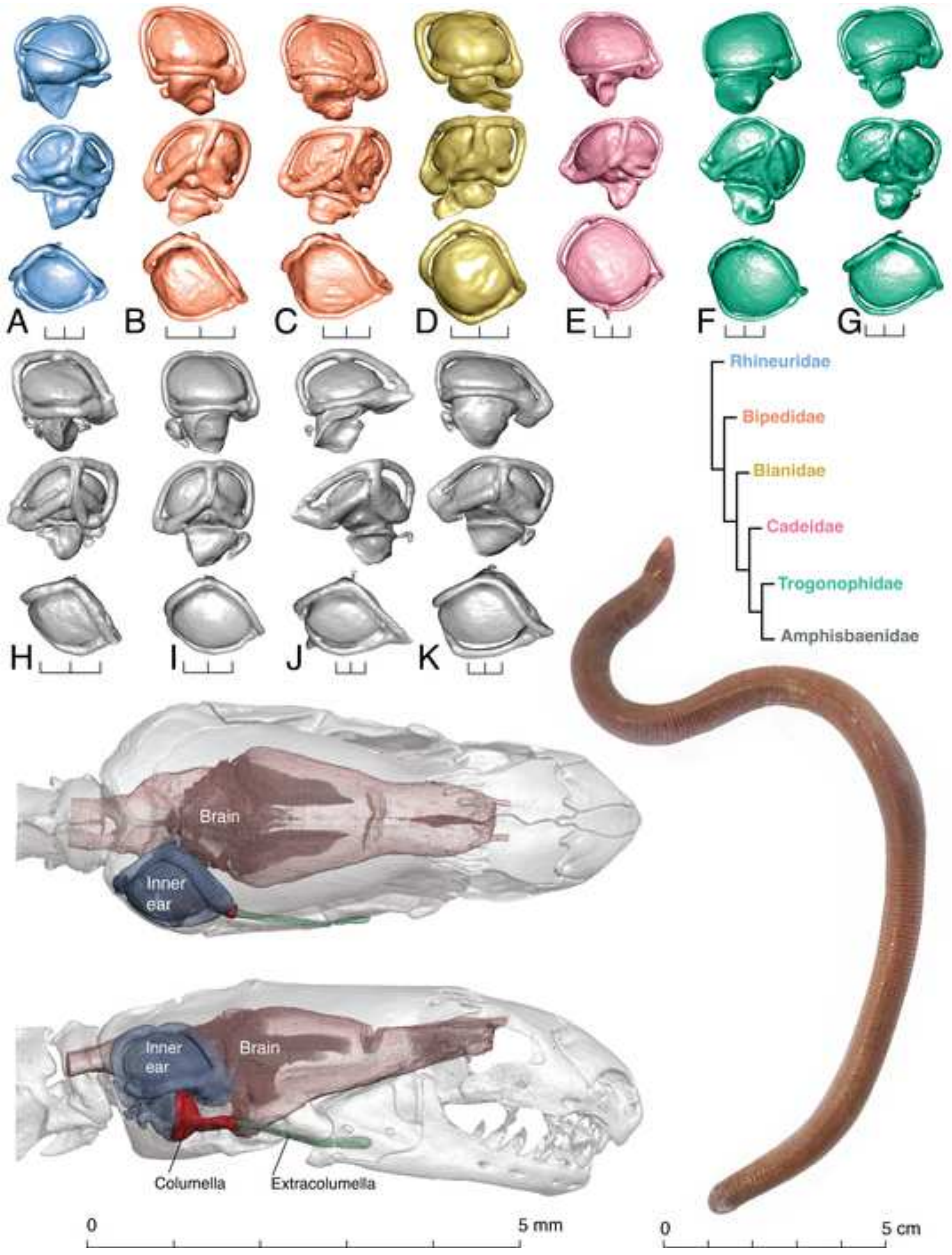

Figure 5

Click here to access/download;Figure;Figure 5.png

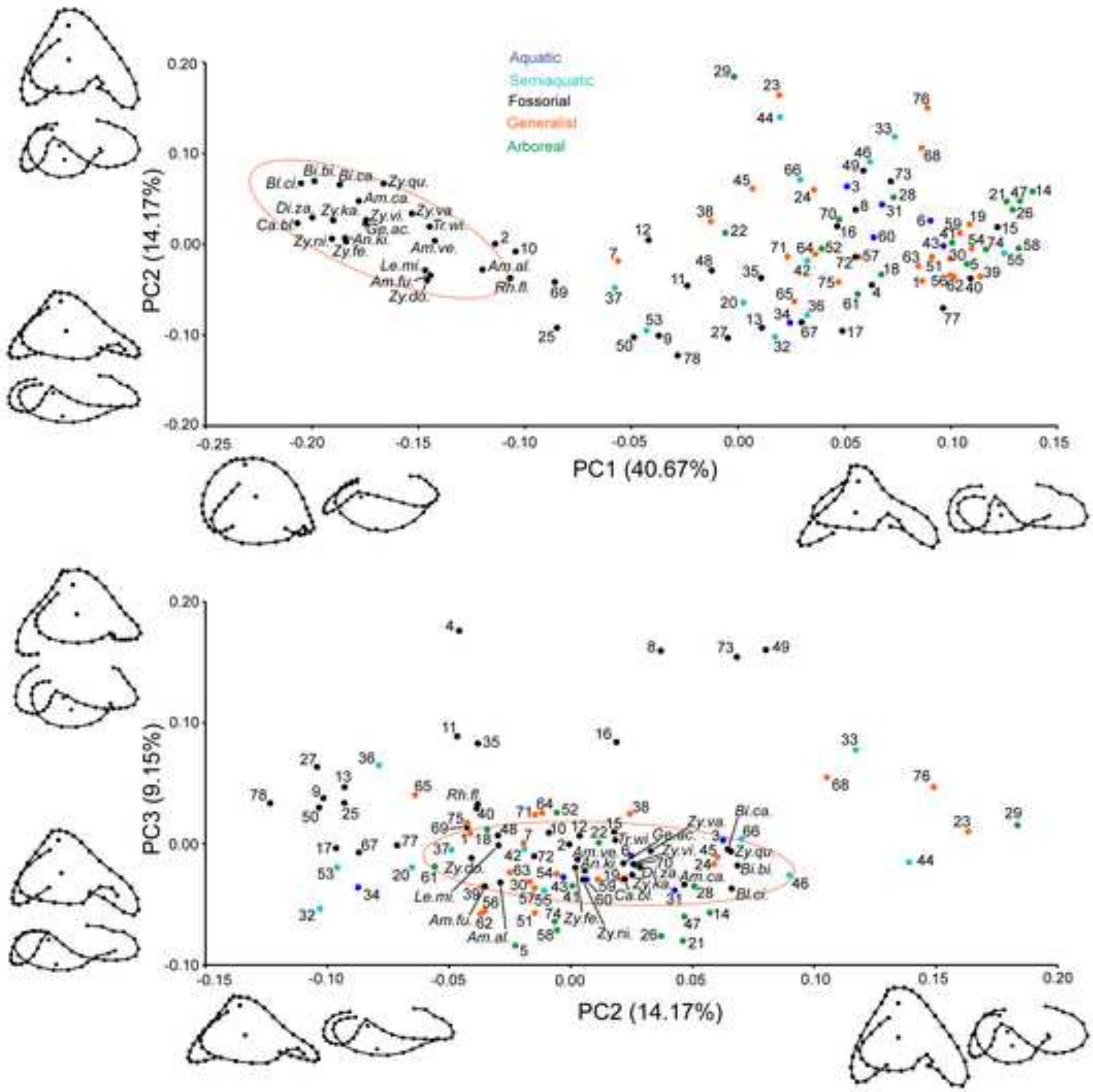

Figure 6

Click here to access/download;Figure;Figure 6.pdf

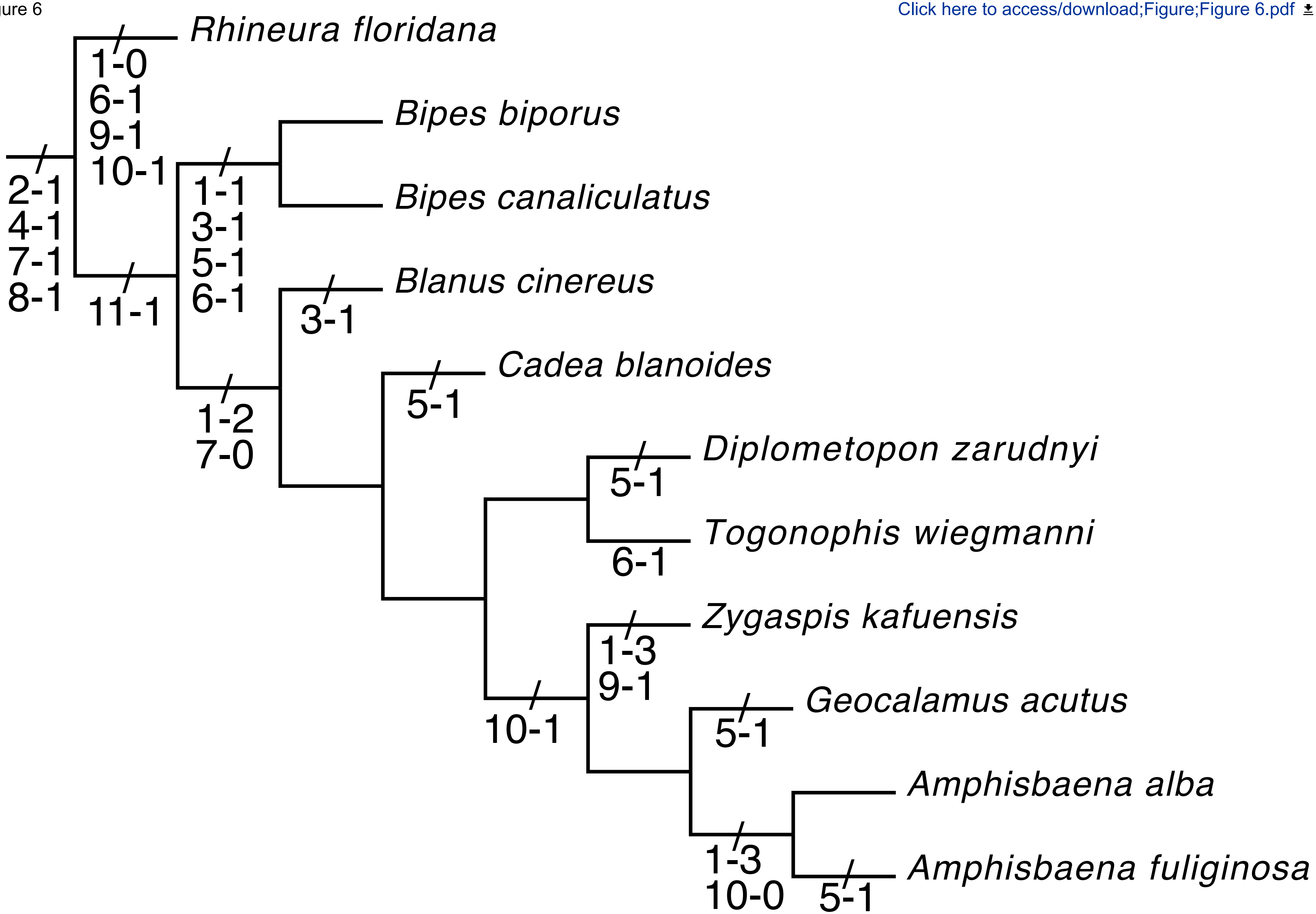

Figure 7

Click here to access/download;Figure;Figure 7.pdf

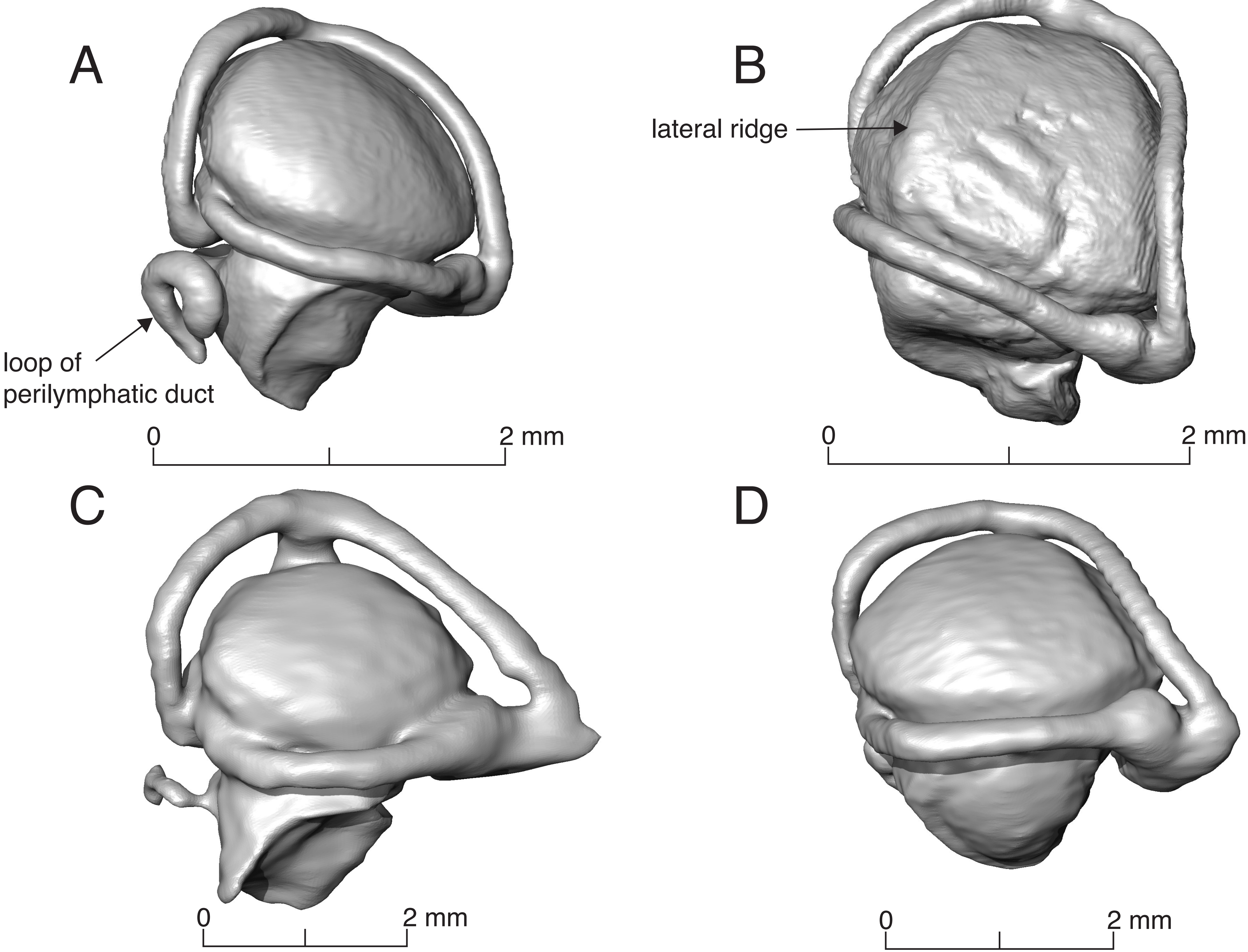

Click here to access/download
Supporting Information
Supporting Information 1 Aligned coordinates copy.txt

Supporting Information 2

Click here to access/download
Supporting Information
Supporting Information 2.xlsx
