# Supplementary material for: The specialized inner ear labyrinth of worm-lizards (Amphisbaenia: Squamata)
Source: PLoS One. 2024 Nov 14;19(11):e0312086. doi: 10.1371/journal.pone.0312086 (PMC11563369; doi:10.1371/journal.pone.0312086)
Supplement: Attachment — Submitted filename: Rebuttal.docx [file pone.0312086.s006.docx]

Dear Dr. Ulrich Joger

Here is my rebuttal to the comments, all my responses are in bold letters. There is also a response to many more comments on the pdf. We remove all the changes indiced by you:

- L. 38 - reptiles
- L. 217 - delete 'in a' (double)
- L. 260 - delete 'skull length'
- L. 407 - potentially
- L. 471 - delete 'useful' (double)
- L. 505 - replace 'not possible decribe' by the previous wording 'difficult to describe'

We also correted a few more typos, indicated in the new marked copy.
